# Adipose-Derived Stromal Cells for Treatment of Patients with Chronic Ischemic Heart Disease (MyStromalCell Trial): A Randomized Placebo-Controlled Study

**DOI:** 10.1155/2017/5237063

**Published:** 2017-12-03

**Authors:** Abbas Ali Qayyum, Anders Bruun Mathiasen, Naja Dam Mygind, Jørgen Tobias Kühl, Erik Jørgensen, Steffen Helqvist, Jens Jørgen Elberg, Klaus Fuglsang Kofoed, Niels Groove Vejlstrup, Anne Fischer-Nielsen, Mandana Haack-Sørensen, Annette Ekblond, Jens Kastrup

**Affiliations:** ^1^Department of Cardiology & Cardiac Catheterization Laboratory 2014, The Heart Centre, Rigshospitalet, University of Copenhagen, Blegdamsvej 9, 2100 Copenhagen, Denmark; ^2^Department of Plastic Surgery, Rigshospitalet, University of Copenhagen, Blegdamsvej 9, 2100 Copenhagen, Denmark; ^3^Department of Radiology, Diagnostic Center, Rigshospitalet, University of Copenhagen, Blegdamsvej 9, 2100 Copenhagen, Denmark; ^4^Department of Clinical Immunology, Rigshospitalet, University of Copenhagen, Blegdamsvej 9, 2100 Copenhagen, Denmark; ^5^Cardiology Stem Cell Centre, The Heart Centre, Rigshospitalet, University of Copenhagen, Blegdamsvej 9, 2100 Copenhagen, Denmark

## Abstract

We aimed to evaluate the effect of intramyocardial injections of autologous VEGF-A_165_-stimulated adipose-derived stromal cells (ASCs) in patients with refractory angina. MyStromalCell trial is a randomized double-blind placebo-controlled study including sixty patients with CCS/NYHA class II-III, left ventricular ejection fraction > 40%, and at least one significant coronary artery stenosis. Patients were treated with ASC or placebo in a 2 : 1 ratio. ASCs from the abdomen were culture expanded and stimulated with VEGF-A_165_. At 6 months follow-up, bicycle exercise tolerance increased significantly in time duration 22 s (95%CI −164 to 208 s) (*P* = 0.034), in watt 4 (95%CI −33 to 41, 0.048), and in METs 0.2 (95%CI −1.4 to 1.8) (*P* = 0.048) in the ASC group while there was a nonsignificant increase in the placebo group in time duration 9 s (95%CI −203 to 221 s) (*P* = 0.053), in watt 7 (95%CI −40 to 54) (*P* = 0.41), and in METs 0.1 (95%CI −1.7 to 1.9) (*P* = 0.757). The difference between the groups was not significant (*P* = 0.680, *P* = 0.608, and *P* = 0.720 for time duration, watt, and METs, resp.). Intramyocardial delivered VEGF-A_165_-stimulated ASC treatment was safe but did not improve exercise capacity compared to placebo. However, exercise capacity increased in the ASC but not in the placebo group. This trial is registered with ClinicalTrials.gov NCT01449032.

## 1. Introduction

Ischemic heart disease is one of the leading causes of premature death worldwide [1]. Due to improvements in medical and interventional therapies at the acute and chronic stages of ischemic heart disease, the years of life lived with chronic ischemic heart disease (CIHD) have increased during the last decades [2]. However, in spite of these improvements, a large number of patients still suffer from refractory angina leading to reduced work capacity and reduced quality-of-life due to impaired myocardial perfusion. To improve myocardial perfusion and thereby cardiac symptoms, stem cell therapy is intensively investigated preclinically and clinically but the optimal cell source has yet to be identified.

Adipose tissue is of mesodermal origin and contains multipotent adipose-derived stromal cells (ASCs), which have the same potential for differentiation and regeneration as bone marrow-derived mesenchymal stromal cells (MSCs) [3–6]. Adipose tissue is available in larger quantities, and the stromal vascular fraction (SVF) obtained contains a much higher concentration of ASCs than MSCs from the bone marrow [5, 7]. As compared to the highly heterogeneous cell population in the SVF isolated immediately after liposuction and used in studies as the Precise and Athena trials [8, 9], the cell population obtained after culture expansion is almost 100% homogeneous ASC population. A higher concentration of ASCs injected into patients with ischemic heart disease may result in a more pronounced treatment [10–13]. ASCs are reported to be more angiogeneic than MSCs, which potentially favors myocardial perfusion and regeneration in CIHD [14–16]. Furthermore, in vitro serum starvation and vascular endothelial growth factor (VEGF) stimulation facilitate differentiation of both MSCs and ASCs towards endothelial cell linage [17, 18]. Therefore, a clinical treatment with ASCs primed towards endothelial cell lineage could potentially be beneficially in inducing angiogenesis.

The MyStromalCell trial is a follow-up to our previous clinical trial, where patients with stable CIHD were treated with equally prestimulated bone marrow MSCs [10].

So far, clinical treatment experience with adipose-derived cells is limited to the adipose-derived SVF, and culture-expanded ASCs have not been tested in patients with ischemic heart disease [8, 9, 19]. The aim of this randomized double-blind placebo-controlled clinical trial was to investigate the treatment effects of intramyocardial delivered VEGF-A_165_-stimulated culture-expanded ASCs in patients with CIHD and refractory angina with preserved left ventricle ejection fraction (LVEF) > 40%.

## 2. Methods

### 2.1. Study Design

MyStromalCell trial is a phase II, first-in-man, single-center, double-blind, randomized, placebo-controlled study of intramyocardial injections of autologous ASCs in patients with CIHD and refractory angina due to at least one occluded or severely stenotic coronary artery and preserved LVEF. This trial was initiated in 2010 and the enrolment was completed in 2014. The study design has been published previously [20].

The study protocol complies with the Declaration of Helsinki and is approved by the Danish National Ethical Committee (02-268856) and Danish Medicines Agency (2612-2867). The study is registered at ClinicalTrials.gov (NCT01449032). The Good Clinical Practice Unit of the Capital Region monitored the study. All patients provided written informed consent.

### 2.2. Study Population

Patients between 30 and 80 years of age with CIHD and refractory angina due to at least one significantly stenosed coronary artery visualized on invasive angiogram without any options for revascularization were considered for inclusion. The patients had to have ischemic symptoms despite optimal tolerable antianginal medication and LVEF > 40%. Inclusion and exclusion criteria are described in Appendix A and Appendix B. The patients were tested on a maximal bicycle exercise tolerance test (ETT) on which they had to stop due to ischemic heart symptoms. Standard bicycle ETT was used, which is recommended by the Danish Society of Cardiology for the detection of myocardial ischemia. The same protocol was used by Friis et al. [10]. The patients had to cycle between 2 and 10 min at baseline to ensure that their performance status was not too poor or too good.

The patients were randomized 2 : 1 to ASC or placebo, in blocks of six with a computer-generated list by an unrelated study person.

### 2.3. Coronary Angiography

Standard clinical techniques were used for coronary angiography. A trained interventional cardiologist blinded to all other data interpreted the angiograms. A coronary diameter stenosis ≥ 70% was considered significant. The patients were discussed by the clinical heart team for standard interventions, and if there were no further options, the patients were considered for inclusion in this study.

### 2.4. Endpoints

The primary outcome was a change in ETT from baseline to 6 months posttreatment. The statistic power was estimated to be more than 90% with an enrolment of 60 patients for detection of an improvement of 60 s (SD assumed to be 35 s) in the treatment group compared to the placebo group.

Secondary endpoints were changes in Canadian Cardiovascular Society (CCS) and New York Heart Association (NYHA) class, Seattle Angina Questionnaire, weekly use of nitroglycerin and weekly frequency of angina attacks 3 and 6 months after the treatment, and myocardial perfusion measured by CT scan at 6 months follow-up.

### 2.5. Cell Harvesting, Culturing, Expansion, and Transplantation

Liposuction was performed in local anesthesia from the subcutaneous abdominal adipose tissue by an experienced plastic surgeon. Between 100 and 150 mL of abdominal adipose tissue was obtained. The process of culturing and expansion of ASCs has been published in detail previously [20]. The lipoaspirate was washed twice with phosphate-buffered saline (PBS) (Gibco, Life Technologies) to remove residual blood. The adipose tissue was digested by collagenase NB6 (Serva GmbH), neutralized with complete medium containing Dulbecco's modified Eagle's medium low glucose 1 g/L (DMEM) (Gibco, Life Technologies), 1% penicillin/streptomycin (10,000 U/mL and 10.000 *μ*g/mL, resp.) (Gibco, Life Technologies), and 10% fetal bovine serum (FBS) (Gamma irradiated, Australian origin, Gibco, Life Technologies) according to a well-established protocol after which the suspension was filtered (Cell Strainer, BD Falcon), centrifuged, and resuspended in complete medium [18]. The isolated SVF was seeded in 75T culture flasks. The complete medium was changed every 3-4 days and passaged when they reached 80–90% confluence. The culture-expanded ASCs were stimulated to differentiate towards endothelial cell linage by culturing in VEGF-A_165_ medium for 7 days before transplantation. The harvested ASCs were suspended in PBS and 0.1% human albumin in a total volume of 3 mL for patients who received active therapy while PBS and 0.1% human albumin were mixed with blood from the patient to a total volume of 3 mL for the placebo treatment. This was blinded to the operator of the intramyocardial injections. The patients allocated to the ASC group were treated with the full amount of ASCs reached after two culture expansion passages. Criteria for release were sterility, viability, and morphology, and the culture media were tested for bacteria, yeast, and mycoplasma 1 week prior to the treatment and on the day of treatment. The ASCs were defined according to criteria established by The International Society for Cellular Therapy (ISCT).

A 3D electromechanical mapping of the left ventricle endocardium was performed using NOGA® system (Biologics Delivery Systems, CA, US) to identify viable and ischemic myocardium. A NOGA Myostar® catheter was used to deliver 10–15 injections of 0.2 mL of ASCs or placebo.

### 2.6. Cardiac CT Acquisition and Analysis

CT scans were performed using a 320-multidetector CT scanner (Aquilion One, Toshiba Medical Systems Corporation, Japan). The CT protocol and the measured parameters have been described in details previously [21, 22]. First, scout images were acquired to localize the heart. Then, rest perfusion images were acquired. Images were acquired from above the origin of the coronary arteries to the diaphragm. Finally, adenosine stress perfusion images were acquired.

Images were reconstructed with 0.5 mm slice thickness and increments of 0.25 mm in 2% interval in the prospective window and transferred to a workstation (Vitrea 6.2 Vital Images Inc., Minnetonka, Minnesota, USA) for analysis.

Attenuation density (AD), perfusion index (PI), and transmural perfusion ratio (TPR) were obtained and used for the semiquantitative analysis after semiautomatically tracing of endo- and epicardial borders of the myocardium. The PI was calculated as the ratio between the myocardial AD divided by AD in the left ventricle blood pool while TPR was calculated as the ratio of AD between endo- and epicardium.

Moreover, left ventricle end-diastolic volume, left ventricle thickness, and left ventricle muscle mass were obtained for further analysis.

### 2.7. Statistics

SPSS version 23.0 (SPSS Inc., Chicago, Illinois) was used for data analysis. Continuous variables are presented as mean ± standard deviation (SD) or 95% confidence interval and categorical variables are presented as numbers and percentages. Paired *t*-test is used for comparison of continuous data within groups while unpaired *t*-test is used for comparison between groups. Repeated measure with autoregressive covariance structure is used for follow-up data with more than two time-points (bicycle ETT, symptoms, angina attacks, use of short-term nitroglycerin, and Seattle Angina Questionnaire). The data were analyzed as intention-to-treat analysis with patients present at follow-up. Categorical data were compared using Fisher's exact test. A two-tailed probability value < 0.05 was considered to indicate statistical significance.

## 3. Results

**Figure 1:**
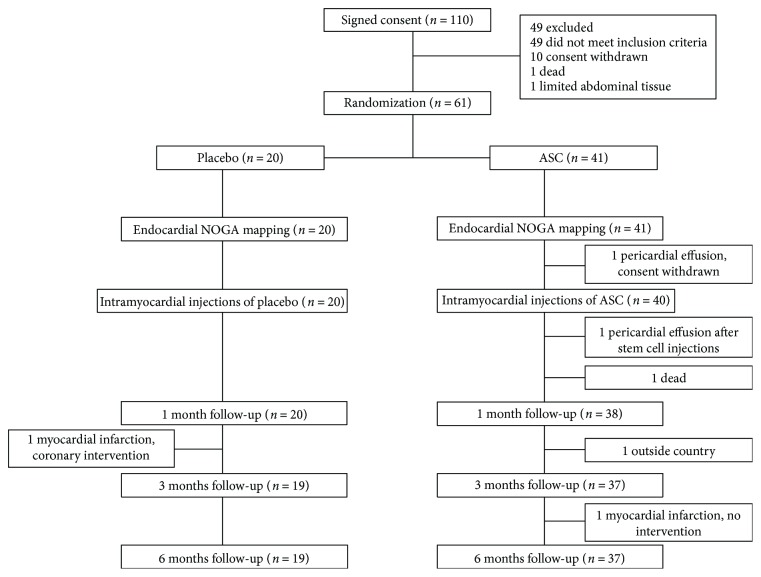
Study design. Eligibility, randomization into adipose-derived stromal cell (ASC) or placebo group, and follow-up.

Sixty-one patients were randomized and sixty patients were treated with either ASCs or placebo (Figure 1). One patient did not show up for 3 months follow-up due to vacation outside the country but returned to attend the 6 months follow-up. Baseline characteristics are shown in Table 1. All patients had previously undergone percutaneous coronary intervention and/or coronary artery bypass grafting. The medication was kept unchanged during the 6 months follow-up period.

The abdominal liposuction volume resulted in 94 ± 25 mL (mean ± SD). The isolated stromal vascular fraction amounted to 94 ± 61 × 10^6^ cells (mean ± SD). The cells were culture expanded for two passages under good manufacturing practice conditions for 32 ± 14 days (mean ± SD) which resulted in 72.0 ± 44.9 × 10^6^ ASCs (mean ± SD) having normal morphology. The cell viability was 89 ± 5% (mean ± SD), and there was no contamination with bacteria, yeast, or mycoplasma.

### 3.1. Serious Adverse Events

One patient had a pericardial effusion during NOGA mapping. The procedure was stopped before injection of stem cells and the patient was treated with pericardiocentesis and withdrew consent. Another patient had pericardial effusion after stem cell injections. The patient was treated with surgical intervention. Both recovered without any sequela.

One patient from the ASC group died before 1-month follow-up due to sudden cardiac arrest. One patient from the ASC group had a transient sinoatrial block, and two patients (ASC and placebo group) had transient nonsustained ventricular tachycardia related to the NOGA procedure. One ASC-treated patient had an allergic reaction observed after the NOGA procedure with rash and fever. In all cases, there were no needs for any treatment. Two patients (from ASC and placebo group) had acute myocardial infarction during 6 months of follow-up. There were no statistical significant differences between the groups (Table 2). No patient had more than 1 event. Figure S1 shows the serial levels of troponin T and CKMB.

### 3.2. Exercise Tolerance Testing

**Figure 2:**
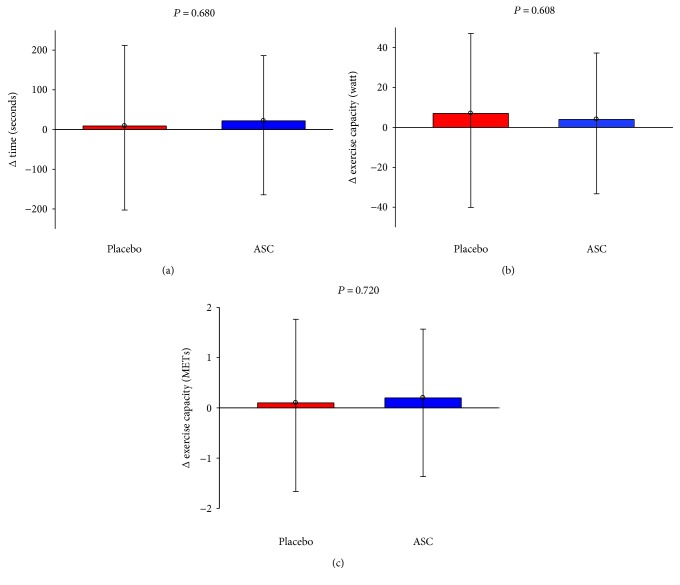
Bicycle exercise test. Between-group comparison of primary endpoint (a) time duration, (b) watt, and (c) metabolic equivalents (METs) of changes from baseline to 6 months follow-up (values are mean ± 95% confidence interval).

**Figure 3:**
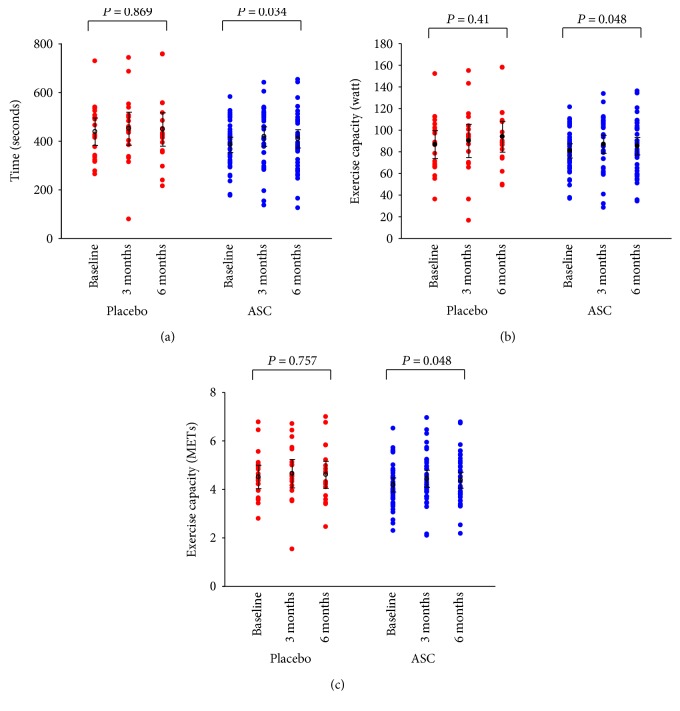
Bicycle exercise test. Primary endpoint (a) time duration, (b) watt, and (c) metabolic equivalents (METs) at baseline, 3, and 6 months follow-up for placebo group and patients treated with adipose-derived stromal cells (ASCs) (values are mean ± 95% confidence interval).

The primary endpoint, changes in ETT from baseline to follow-up, was increased in the placebo group 9 s (95% CI –203 to 221 s) and in the ASC group 22 s (95% CI –164 to 208 s). However, there was no statistical significant difference between the groups (*P* = 0.680) (Figure 2(a)). The mean bicycle ETT duration at baseline was 437 ± 53 s and 383 ± 30 s (*P* = 0.053) for the placebo and ASC groups, respectively. At 6 months follow-up, the ETT duration of time increased to 446 ± 64 s and 407 ± 36 s in placebo group and ASC group, respectively. The increase in time duration, from baseline to follow-up, was only significant in the ASC group (*P* = 0.034) (Figure 3(a)).

There was no difference between the two groups for change in watt from baseline to 6 months follow-up (7 (95% CI –40 to 54) watt and 4 (95% CI –33 to 41) watt; *P* = 0.608 for the placebo group and for the ASC group, resp.) (Figure 2(b)). The ASC group performed 81 ± 6 watts at baseline which increased significantly to 85 ± 8 watts at 6 months follow-up (*P* = 0.048). There was no significant change in the placebo group (87 ± 12 watts at baseline and 94 ± 13 watts at 6 months follow-up, *P* = 0.41) (Figure 3(b)).

The metabolic equivalents (METs) performed by the patients in the ASC group did also increase significantly from 4.2 ± 0.3 at baseline to 4.4 ± 0.3 at 6 months follow-up (*P* = 0.048), while it was unchanged in the placebo group (4.5 ± 0.4 at baseline and 4.6 ± 0.5 at follow-up, *P* = 0.757) (Figure 3(c)). However, there was no difference between the two groups for change in METs from baseline to 6 months follow-up (0.1 (95% CI –1.7 to 1.9) and 0.2 (95% CI –1.4 to 1.8); *P* = 0.720 for the placebo group and for the ASC group, resp.) (Figure 2(c)).

### 3.3. Symptoms and Antianginal Medication

**Figure 4:**
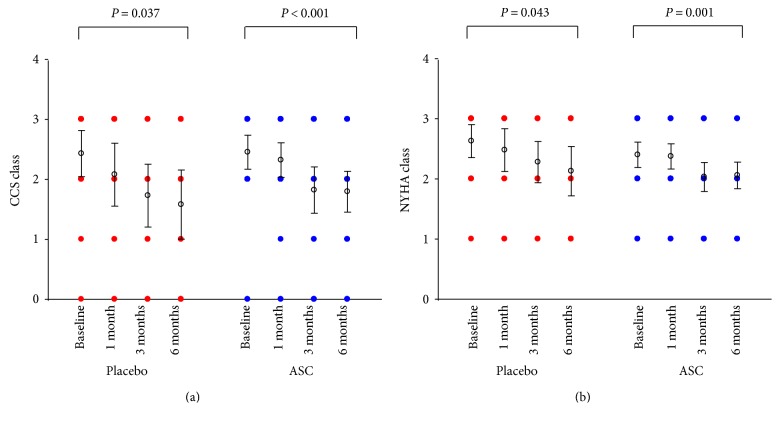
Symptoms measured as functional classes. (a) Canadian Cardiovascular Society (CCS) and (b) New York Heart Association (NYHA) (values are mean ± 95% confidence interval).

All patients had at least CCS class II and/or angina-equivalent dyspnea NYHA class II at baseline. During the 6-month follow-up time, there was a significant decrease in CCS class for patients both in the placebo and ASC group, *P* = 0.037 and *P* < 0.001, respectively (Figure 4(a)). There was a similar decrease in NYHA class for placebo and ASC groups, *P* = 0.043 and *P* = 0.001, respectively (Figure 4(b)).

There was a significant decrease in weekly angina attacks for both the patients in the placebo and ASC groups (*P* = 0.032 and *P* = 0.002, resp.) (Figure S2A). However, the use of short-term nitroglycerin was not significantly reduced in any of the groups (Figure S2B).

### 3.4. Seattle Angina Questionnaire

There was a significant improved angina stability score, angina frequency score, quality-of-life, and physical limitation score in both groups (Figure S3). Nevertheless, there was no change in overall satisfaction score in the groups. No significant differences between the two groups were observed for Seattle Angina Questionnaire.

### 3.5. CT Perfusion Images and Functional Analysis

All patients underwent rest CT scan and 54 patients went through stress CT scan at baseline. At 6 months follow-up, 56 patients underwent rest CT scan while 49 patients had a stress CT scan performed. There were no differences between the groups for global attenuation density (AD), perfusion index (PI), or transmural perfusion ratio (TPR) (Table 3(a)). Additionally, there were no significant differences between the two groups in AD, PI, or TPR related to the coronary territories where the injections were performed. Moreover, there were no differences for left ventricle end-diastolic volume, left ventricle thickness, or left ventricle muscle mass (Table 3(b)).

## 4. Discussion

MystromalCell trial is the first-in-man randomized, double-blind placebo-controlled study using VEGF-A_165_-stimulated culture-expanded ASCs for patients with refractory angina due to CIHD and preserved LVEF. There were no statistical significant differences between the groups for the primary endpoint, change in the bicycle exercise tolerance test, from baseline to 6 months follow-up. However, there was a significant increase in bicycle exercise tolerance test in time duration and work capacity in patients receiving ASCs, which was not seen in the placebo group. A significant improvement in CCS class and NYHA class in the ASC and placebo group was also observed. Furthermore, there was a significant decrease in weekly angina attacks and improved quality-of-life score in both groups.

The aim of this study was to improve exercise capacity due to increased myocardial perfusion induced by VEGF-A_165_-stimulated ASCs. Using static CT perfusion analysis, we did not detect any significant change in global myocardial perfusion during rest or pharmacological stress. Although rest and stress AD decreased slightly from baseline to follow-up in the placebo group and remained unchanged in the ASC group, the changes may have been too small to be detected by CT on either global or vessel territorial level or the analysis method may need to be optimized. Furthermore, this imaging method is not designed to register absolute changes in microvascular perfusion but only relative changes.

Another treatment method for improvement of myocardial perfusion using transmyocardial laser revascularization has been tested in several clinical trials but without any clear effect [23]. However, we cannot exclude that the intramyocardial injections by itself improve the myocardial perfusion independently of the stem cells.

The finding that the weekly use of nitroglycerin was unchanged while weekly angina attacks decreased along with an increasing angina frequency score is an indication of increasing health status and therefore in accordance with the improved clinical status observed.

The improvement in exercise tolerance and in angina observed in this study is consistent with the previous published reports using exercise test as an outcome measure after CD34^+^ treatment in patients with refractory angina [24, 25] while another study showed a greater increase in exercise time than observed in this study [26]. These studies might have used different protocols so a direct comparison of the exercise tolerance has to be done carefully. However, with a 2 : 1 ratio and the present population size, we cannot reject a type 2 error in this study.

Preclinical studies have previously demonstrated the benefit of intramyocardial injection of ASCs. In mice with CIHD, ASC improved LVEF assessed by echocardiography and ^18^F-FDG microPET imaging [27]. Moreover, intramyocardially injected ASCs have demonstrated increased LVEF, wall thickness, and reduction of infarct size in rats [28].

A small study using an intramyocardial injection of freshly harvested adipose-derived SVF cells in patients with refractory angina showed that exercise capacity measured as METs in the active group remained stable while there was a decrease in METs in the placebo group [9]. Another small study delivering freshly harvested adipose-derived SVF cells intracoronary in patients with ST-elevation myocardial infarction showed a trend towards improved LVEF [19]. The Athena trials used an intramyocardial injection of adipose-derived SVF cells in patients with ischemic heart failure and showed that maximum oxygen consumption on exercise treadmill testing was increased in the therapy group but not significantly different from the placebo group [8]. Used SVF cell populations are however heterogeneous, compromising approximately 2% ASCs [29]. To achieve sufficient numbers of SVF effector cells for treatment without culture expansion, 2-3 times the amount of fat tissue has to be harvested from the patients [8].

A change in paradigm has occurred in the understanding of the regenerative mechanisms of ASCs since the initiation of MyStromalCell. As such, the decisive factor for the regenerative capacity of ASCs is now believed to be of paracrine nature, and therefore, an angiogeneic prestimulation protocol as of the present is potentially not needed [29].

The ADVANCE study was a phase II double-blinded safety trial in Europe which planned to enroll 360 patients with acute myocardial infarction to be randomized either to intracoronary delivered freshly harvested adipose-derived SVF cells or placebo. The preliminary data showed the safety of the treatment and the study group terminated the trial before full enrolment [30].

Previously, bone marrow-derived MSCs have shown to reduce symptoms of patients with CIHD with and without heart failure [10, 12, 13].

In 50 patients with CIHD randomized to either intramyocardial injections of bone marrow-derived mononuclear cells or placebo, cell therapy demonstrated improved myocardial perfusion assessed by single-photon emission computed tomography 3 months after the treatment [31].

A significant difference between the active and the placebo groups in the primary endpoint, changes in ETT from baseline to follow-up between the groups, was not reached. The power calculation prior to the study start estimated a difference in time duration between the groups of 60 s [20]. However, the difference in time duration was less than half of the expected amount. A power calculation with the present standard deviation of 24 s, a power of 90%, and a randomization ratio of 2 : 1 with a significance level of 5% indicates that twice the patient population included into this study is needed to detect the observed difference in the change of time between the two groups. Probably, the number of enrolled patients has to be even higher due to patient dropout, death, withdrawal of consent, and so forth. Moreover, an increase of at least 0.5 METs may be of value for the patient [32]. In upcoming randomized trials, a combined endpoint could be a more suitable endpoint.

The patients were referred from other hospitals and coronary angiography databases were looked through to find potential candidates to be included into this study. However, these no-option patients may be more difficult to find today since new techniques for interventions on chronic total coronary occlusions and so forth are now more widely implemented.

Most clinical studies as of the present use autologous stem cells. However, with this design, it is difficult to standardize the treatment. The ASCs have to be culture expanded for several weeks and the number and quality of cells reached for treatment vary considerably from patient to patient. This could potentially have had an impact on the effect of the therapy in this study. With newly established cell culture methods using closed bioreactor systems and optimized cultivation media, a much higher cell yield can be reached [33]. Therefore, it can be speculated whether this technology and a standardized allogeneic stem cell product from healthy donors together with a higher cell number for treatment would have had improved the clinical outcome.

Preconditioning or genetically modifying the stem cells, before transplantation, may play a role and have to be investigated further. Moreover, one injection or multiple injections, route of cell delivery, the optimal cell source, and the process during culture expansion are some of the steps, which may have importance for the eventual effect. However, we still need larger studies and international multicenter studies, such as the SCIENCE trial (NCT02673164), which may be the next step forward in order to establish stem cell therapy as a daily practice therapy outside controlled trials. At the moment, stem cell therapy should only be performed in controlled trials. Nevertheless, the present study is still an important report due to the safety and feasibility demonstration. It should be emphasized that myocardial perforation caused by the NOGA mapping procedure and the intramyocardial injections calls for highly experienced operators to minimize the risk of this serious complication.

Compared to conventional medical and interventional therapy, cell-based therapies have the potential to regenerate the ischemic myocardium [34]. However, based on the present and previous results, it can be concluded that to detect a significant difference in exercise tolerance between the placebo and the present used stem cell, several hundreds of patients have to be enrolled [35].

Limitations of the study are that at the baseline; the exercise time duration is seemingly better in the placebo group compared to the ASC group and the changes seem like they are increasing constantly. Some of the patients were lost to follow-up, and there was an imbalance in some of the measurements between the groups. Moreover, the observed effect sizes were smaller than probably clinical relevant and the groups are relatively small. The increased performance on bicycle ETT could be due to the fact that the patients got used to performing the test. It will be interesting in the future to follow these patients to observe how their symptoms and work capacity changes and whether the placebo effect disappears.

In conclusion, this double-blind placebo-controlled study using VEGF-A_165_-stimulated culture-expanded autologous ASCs demonstrates that the treatment was safe but did not show a difference between the groups in the primary endpoint ETT. However, there was an increase in the ASC group for the ETT, which was not seen in the placebo group.

## Figures and Tables

**Table 1:** Baseline characteristics.

Parameter	Placebo (*n* = 20)	ASC (*n* = 40)	*P* value
Age (years)	65.3 ± 8.7	65.5 ± 9.7	0.94
Male gender	20 (100)	35 (87.5)	0.02
BMI (kg/m^2^)	30.0 ± 4.8	30.0 ± 4.1	0.92
*Smoking*			0.19
Current	3 (15)	8 (20)	
Previous	16 (80)	23 (57.5)	
Never	1 (5)	9 (22.5)	
Diabetes mellitus	6 (30)	16 (40)	0.57
Hypertension	12 (60)	33 (82.5)	0.06
AMI	10 (50)	26 (65)	0.26
CABG	20 (100)	33 (82.5)	0.08
PCI	15 (75)	28 (70)	0.69
LVEF (%)	54 ± 8	52 ± 8	0.38
Systolic blood pressure (mmHg)	133 ± 19	132 ± 17	0.91
Diastolic blood pressure (mmHg)	76 ± 11	76 ± 12	0.99
Heart rate (beats/min)	66 ± 9	63 ± 7	0.14
FEV1 (L)	2.27 ± 0.61	2.34 ± 0.69	0.69
FVC (L)	3.17 ± 0.65	3.11 ± 0.80	0.76
FEV1/FVC	71.2 ± 10.3	74.8 ± 7.7	0.18
Pro-BNP (pmol/L)	30.4 ± 24.3	39.6 ± 37.5	0.27
TnT (ng/L)	12.5 ± 2.1	14.9 ± 7.8	0.09
CK-MB (*μ*g/L)	2.8 ± 1.6	3.2 ± 1.4	0.36
Hgb A1c (mmol/L)	6.1 ± 0.6	6.7 ± 1.3	0.03
Total cholesterol (mmol/L)	3.8 ± 0.7	4.3 ± 1.2	0.06
HDL-C (mmol/L)	1.0 ± 0.2	1.2 ± 0.4	0.05
LDL-C (mmol/L)	1.9 ± 0.6	2.5 ± 1.0	0.02
Triglycerides (mmol/L)	2.6 ± 1.5	1.8 ± 0.8	0.06
CRP (mg/L)	5.0 ± 10.0	3.6 ± 3.7	0.57
*Medication*			
Acetylsalicylic acid	19 (95)	35 (87.5)	0.65
Clopidogrel	7 (35)	11 (27.5)	0.56
ACE-I or ARB	13 (65)	29 (72.5)	0.42
*β*-Blocker	16 (80)	33 (82.5)	1.00
Calcium antagonist	12 (60)	19 (47.5)	0.42
Diuretics	12 (60)	27 (67.5)	0.58
Statins	20 (100)	40 (100)	1.00
Nitrate	19 (95)	28 (70)	0.04
Nicorandil	6 (30)	5 (12.5)	0.16
Ivabradine	2 (10)	4 (10)	1.00

ACE-I: angiotensin-converting enzyme inhibitor; AMI: acute myocardial infarction; ARB: angiotensin II receptor blockers; ASC: adipose-derived stromal cell; BMI: body mass index; CABG: coronary artery bypass grafting; CK-MB: creatine kinase MB; CRP: C-reactive protein; FEV1: forced expiratory volume in 1 s; FVC: forced vital capacity; HDL-C: high-density lipoprotein cholesterol; Hgb A1c: hemoglobin A1c; LDL: low-density lipoprotein cholesterol; LVEF: left ventricular ejection fraction; *n*: number of patients; PCI: percutaneous coronary intervention; Pro-BNP: probrain natriuretic peptide; TnT: troponin T.

**(a):** 

Serious adverse events	Placebo (*n* = 20)	ASC (*n* = 40)	*P* value
Death	0 (0)	1 (2.5)	1.00
Hospitalizations			
Myocardial infarction	1 (5)	1 (2.5)	1.00
Dyspnea	0 (0)	1 (2.5)	1.00
Anemia	1 (5)	1 (2.5)	1.00
Syncope	1 (5)	0 (0)	0.33
Peripheral edema	0 (0)	1 (2.5)	1.00
Angina worsening	3 (15)	6 (15)	1.00
Pneumonia	1 (5)	1 (2.5)	1.00
NOGA-related complications			
Pericardial effusion	0 (0)	1 (2.5)	1.00
ECG changes—SA-block, NsVT	2 (10)	1 (2.5)	0.26
Hematoma at femoral puncture	1 (5)	0 (0)	0.33
Allergic reaction	0 (0)	1 (2.5)	1.00
Bradycardia	0 (0)	1 (2.5)	1.00
General discomfort	0 (0)	1 (2.5)	1.00

NsVT: nonsustained ventricular tachycardia; SA-block: sinoatrial block. Values are *n* (%); *P* values are calculated using Fischer's exact test.

**(b):** 

	Placebo (*n* = 20)	ASC (*n* = 40)	*P* value
Major adverse events	4 (20)	9 (22.5)	1.00
Nonmajor adverse events	4 (20)	4 (10)	0.42

Values are *n* (%); *P* values are calculated using Fischer's exact test.

**(a):** 

	Placebo group						ASC						*P* value (differences between groups for rest values)	*P* value (differences between groups for stress values)
	Rest			Stress			Rest			Stress				
	Baseline	Follow-up	*P* value	Baseline	Follow-up	*P* value	Baseline	Follow-up	*P* value	Baseline	Follow-up	*P* value		
AD	92.9 ± 16.0	85.4 ± 16.0	0.132	118.4 ± 17.8	116.1 ± 15.3	0.583	86.2 ± 14.5	86.0 ± 14.7	0.91	118.7 ± 18.0	117.6 ± 20.0	0.675	0.173	0.803
PI	0.20 ± 0.04	0.23 ± 0.13	0.455	0.35 ± 0.05	0.34 ± 0.09	0.792	0.19 ± 0.04	0.19 ± 0.08	0.612	0.33 ± 0.06	0.34 ± 0.07	0.28	0.542	0.419
TPR	1.15 ± 0.13	1.15 ± 0.13	0.959	1.00 ± 0.06	1.05 ± 0.11	0.25	1.15 ± 0.07	1.14 ± 0.10	0.655	1.02 ± 0.06	1.03 ± 0.05	0.733	0.828	0.171

Values are mean ± SD.

**(b):** 

	Placebo			ASC			
	Baseline	Follow-up	*P* value	Baseline	Follow-up	*P* value	*P* value (between groups for differences)
LV EDV	127.1 ± 41.9	135.0 ± 41.4	0.203	139.0 ± 40.1	135.0 ± 40.1	0.37	0.126
LV thickness	8.2 ± 2.4	8.5 ± 2.0	0.327	7.8 ± 1.2	8.2 ± 1.7	0.107	0.923
LV muscle mass	151.5 ± 45.8	157.4 ± 56.0	0.214	143.1 ± 34.4	139.6 ± 31.3	0.449	0.199

Values are mean ± SD

## Appendix

### A. Inclusion criteria

- Age between 30 and 80 years
- Moderate to severe angina (CCS angina classes II-III) or angina equivalent dyspnea (NYHA classes II-III) despite optimal medical therapy
- Must have, within 12 months prior to entry, documented coronary angiographic evidence of significant vessel disease and at least one remaining larger coronary vessel from which new collaterals/vessels could be supplied
- Must not be eligible for any other revascularization procedures
- Left ventricular ejection fraction > 40% measured by echocardiography, SPECT, CT-scan, or MRI
- Duration of bicycle ergometry exercise tolerance tests: 2 to 10 minutes
- CABG or PCI within 6 months of entry must have angiography performed at least 4 months after the previous intervention to rule out early restenosis and to document remaining significant vessel disease
- Ventricular wall thickness of the treatment zone > 7 mm measured by echocardiography, CT-scan, or MRI

*Note*. CABG: coronary artery bypass grafting; CCS: Canadian Cardiovascular Society; CT: computed tomography; MRI: magnetic resonance imaging; NYHA: New York Heart Association; PCI: percutaneous coronary intervention; SPECT: single-photon emission computed tomography.

### B. Exclusion criteria

- Pregnant or lactating women
- Clinically significant anemia, leukopenia, leukocytosis, or thrombocytopenia
- Conditions other than angina that will limit exercise test (e.g., severe peripheral vascular disease, COPD, and FEV_1_ < 1)
- Immunocompromised status or currently receiving immunosuppressive therapy
- Valvular heart disease requiring surgical intervention
- Less than 6 weeks prior to screening: ACS with increase in CK-MB or troponins/PCI/CABG/stroke or TIA
- History of malignancy < 5 years (except cured nonmelanoma skin cancer) or suspicion of current malignancy
- Other experimental medications within the last four weeks prior to the baseline ETT

*Note.* ACS: acute coronary syndrome; CABG: coronary artery bypass grafting; COPD: chronic obstructive pulmonary disease; ETT: exercise tolerance test; FEV_1_: forced expiratory volume in one second; PCI: percutaneous coronary intervention; TIA: transient ischemic attack.

## Supplementary Materials

Supplementary files Figure S1: TNT and CKMB levels before and after treatment: (A) TNT and (B) CKMB. (Boxed values are mean ± 95% confidence interval.) Figure S2: Weekly (A) angina attacks and (B) use of short-term nitroglycerin (NTG). (Values are mean ± 95% confidence interval.) Figure S3: Seattle Angina Questionnaires. The (A) angina stability score, (B) angina frequency score, (C) quality of life score, (D) physical limitation score, and (E) satisfaction score (Values are mean ± 95% confidence interval.)

## References

[1] Finegold J. A., Asaria P., Francis D. P. (2013). Mortality from ischaemic heart disease by country, region, and age: statistics from World Health Organisation and United Nations. *International Journal of Cardiology*.

[2] Moran A. E., Forouzanfar M. H., Roth G. A. (2014). The global burden of ischemic heart disease in 1990 and 2010: the global burden of disease 2010 study. *Circulation*.

[3] Strem B. M., Hicok K. C., Zhu M. (2005). Multipotential differentiation of adipose tissue-derived stem cells. *The Keio Journal of Medicine*.

[4] Bochev I., Elmadjian G., Kyurkchiev D. (2008). Mesenchymal stem cells from human bone marrow or adipose tissue differently modulate mitogen-stimulated B-cell immunoglobulin production in vitro. *Cell Biology International*.

[5] Fraser J. K., Schreiber R., Strem B. (2006). Plasticity of human adipose stem cells toward endothelial cells and cardiomyocytes. *Nature Clinical Practice Cardiovascular Medicine*.

[6] Heydarkhan-Hagvall S., Schenke-Layland K., Yang J. Q. (2008). Human adipose stem cells: a potential cell source for cardiovascular tissue engineering. *Cells, Tissues, Organs*.

[7] Helder M. N., Knippenberg M., Klein-Nulend J., Wuisman P. I. J. M. (2007). Stem cells from adipose tissue allow challenging new concepts for regenerative medicine. *Tissue Engineering*.

[8] Henry T. D., Pepine C. J., Lambert C. R. (2017). The Athena trials: autologous adipose-derived regenerative cells for refractory chronic myocardial ischemia with left ventricular dysfunction. *Catheterization and Cardiovascular Interventions*.

[9] Perin E. C., Sanz-Ruiz R., Sanchez P. L. (2014). Adipose-derived regenerative cells in patients with ischemic cardiomyopathy: the PRECISE trial. *American Heart Journal*.

[10] Friis T., Haack-Sorensen M., Mathiasen A. B. (2011). Mesenchymal stromal cell derived endothelial progenitor treatment in patients with refractory angina. *Scandinavian Cardiovascular Journal*.

[11] Hare J. M., Fishman J. E., Gerstenblith G. (2012). Comparison of allogeneic vs autologous bone marrow-derived mesenchymal stem cells delivered by transendocardial injection in patients with ischemic cardiomyopathy: the POSEIDON randomized trial. *JAMA*.

[12] Mathiasen A. B., Haack-Sorensen M., Jorgensen E., Kastrup J. (2013). Autotransplantation of mesenchymal stromal cells from bone-marrow to heart in patients with severe stable coronary artery disease and refractory angina - final 3-year follow-up. *International Journal of Cardiology*.

[13] Mathiasen A. B., Qayyum A. A., Jorgensen E. (2015). Bone marrow-derived mesenchymal stromal cell treatment in patients with severe ischaemic heart failure: a randomized placebo-controlled trial (MSC-HF trial). *European Heart Journal*.

[14] Kim Y., Kim H., Cho H., Bae Y., Suh K., Jung J. (2007). Direct comparison of human mesenchymal stem cells derived from adipose tissues and bone marrow in mediating neovascularization in response to vascular ischemia. *Cellular Physiology and Biochemistry*.

[15] Poncelet A. J., Hiel A. L., Vercruysse J., Hermans D., Zech F., Gianello P. (2010). Intracardiac allogeneic mesenchymal stem cell transplantation elicits neo-angiogenesis in a fully immunocompetent ischaemic swine model. *European Journal of Cardio-Thoracic Surgery*.

[16] Str ioga M., Viswanathan S., Darinskas A., Slaby O., Michalek J. (2012). Same or not the same? Comparison of adipose tissue-derived versus bone marrow-derived mesenchymal stem and stromal cells. *Stem Cells and Development*.

[17] Oswald J., Boxberger S., Jorgensen B. (2004). Mesenchymal stem cells can be differentiated into endothelial cells in vitro. *Stem Cells*.

[18] Follin B., Tratwal J., Haack-Sorensen M., Elberg J. J., Kastrup J., Ekblond A. (2013). Identical effects of VEGF and serum-deprivation on phenotype and function of adipose-derived stromal cells from healthy donors and patients with ischemic heart disease. *Journal of Translational Medicine*.

[19] Houtgraaf J. H., den Dekker W. K., van Dalen B. M. (2012). First experience in humans using adipose tissue-derived regenerative cells in the treatment of patients with ST-segment elevation myocardial infarction. *Journal of the American College of Cardiology*.

[20] Qayyum A. A., Haack-Sorensen M., Mathiasen A. B., Jørgensen E., Ekblond A., Kastrup J. (2012). Adipose-derived mesenchymal stromal cells for chronic myocardial ischemia (MyStromalCell Trial): study design. *Regenerative Medicine*.

[21] Qayyum A. A., Kuhl J. T., Kjaer A., Hasbak P., Kofoed K. F., Kastrup J. (2015). Semi-quantitative myocardial perfusion measured by computed tomography in patients with refractory angina: a head-to-head comparison with quantitative rubidium-82 positron emission tomography as reference. *Clinical Physiology and Functional Imaging*.

[22] Qayyum A. A., Kuhl J. T., Mathiasen A. B. (2013). Value of cardiac 320-multidetector computed tomography and cardiac magnetic resonance imaging for assessment of myocardial perfusion defects in patients with known chronic ischemic heart disease. *The International Journal of Cardiovascular Imaging*.

[23] Briones E., Lacalle J. R., Marin-Leon I., Rueda J. R. (2015). Transmyocardial laser revascularization versus medical therapy for refractory angina. *Cochrane Database of Systematic Reviews*.

[24] Losordo D. W., Schatz R. A., White C. J. (2007). Intramyocardial transplantation of autologous CD34^+^ stem cells for intractable angina: a phase I/IIa double-blind, randomized controlled trial. *Circulation*.

[25] Povsic T. J., Henry T. D., Traverse J. H. (2016). The RENEW trial: efficacy and safety of intramyocardial autologous CD34^+^ cell administration in patients with refractory angina. *JACC: Cardiovascular Interventions*.

[26] Losordo D. W., Henry T. D., Davidson C. (2011). Intramyocardial, autologous CD34^+^ cell therapy for refractory angina. *Circulation Research*.

[27] Mazo M., Planat-Benard V., Abizanda G. (2008). Transplantation of adipose derived stromal cells is associated with functional improvement in a rat model of chronic myocardial infarction. *European Journal of Heart Failure*.

[28] Wang L., Deng J., Tian W. (2009). Adipose-derived stem cells are an effective cell candidate for treatment of heart failure: an MR imaging study of rat hearts. *American Journal of Physiology Heart and Circulatory Physiology*.

[29] Bai X., Alt E. (2010). Myocardial regeneration potential of adipose tissue-derived stem cells. *Biochemical and Biophysical Research Communications*.

[30] March 2016, http://ir.cytori.com/investor-relations/News/news-details/2013/Cytori-Reports-First-Half-and-2nd-Quarter-2013-Business-and-Financial-Results/default.aspx

[31] van R. J., Bax J. J., Beeres S. L. (2009). Intramyocardial bone marrow cell injection for chronic myocardial ischemia: a randomized controlled trial. *JAMA*.

[32] Diderholm E., Andrén B., Frostfeldt G. (2003). Effects of an early invasive strategy on ischemia and exercise tolerance among patients with unstable coronary artery disease. *The American Journal of Medicine*.

[33] Haack-Sorensen M., Follin B., Juhl M. (2016). Culture expansion of adipose derived stromal cells. A closed automated quantum cell expansion system compared with manual flask-based culture. *Journal of Translational Medicine*.

[34] Tongers J., Losordo D. W., Landmesser U. (2011). Stem and progenitor cell-based therapy in ischaemic heart disease: promise, uncertainties, and challenges. *European Heart Journal*.

[35] Povsic T. J., Junge C., Nada A. (2013). A phase 3, randomized, double-blinded, active-controlled, unblinded standard of care study assessing the efficacy and safety of intramyocardial autologous CD34+ cell administration in patients with refractory angina: design of the RENEW study. *American Heart Journal*.

