# Supplementary material for: Adipose-Derived Stromal Cells for Treatment of Patients with Chronic Ischemic Heart Disease (MyStromalCell Trial): A Randomized Placebo-Controlled Study
Source: Stem Cells Int. 2017 Dec 3;2017:5237063. doi: 10.1155/2017/5237063 (PMC5733128; doi:10.1155/2017/5237063)
Supplement: Supplementary files — Figure S1: TNT and CKMB levels before and after treatment: (A) TNT and (B) CKMB. (Boxed values are mean ± 95% confidence interval.) Figure S2: Weekly (A) angina attacks and (B) use of short-term nitroglycerin (NTG). (Values are mean ± 95% confidence interval.) Figure S3: Seattle Angina Questionnaires. The (A) angina stability score, (B) angina frequency score, (C) quality of life score, (D) physical limitation score, and (E) satisfaction score (Values are mean ± 95% confidence interval.) [file 5237063.f1.docx]

**Figure Legends**

**Figure S1** TNT and CKMB levels before and after treatment: A) TNT and B) CKMB. (Box values are mean ± 95% confidence interval).

**Figure S2** Weekly (A) angina attacks and (B) use of short-term nitroglycerin (NTG). (Values are mean ± 95% confidence interval).

**Figure S3** Seattle Angina Questionnaires. The (A) angina stability score, (B) angina frequency score, (C) quality of life score, (D) physical limitation score and (E) satisfaction score. (Values are mean ± 95% confidence interval).

**Figures**

**Figure S1**

A

B

**Figure S2**

A

B

**Figure S3**

A

B

C

D

E
